# Complete Scene Recovery and Terrain Classification in Textured Terrain Meshes

**DOI:** 10.3390/s120811221

**Published:** 2012-08-13

**Authors:** Wei Song, Kyungeun Cho, Kyhyun Um, Chee Sun Won, Sungdae Sim

**Affiliations:** 1 Department of Multimedia Engineering, Dongguk University-Seoul, 26 Pildosng 3 Ga, Jung-gu, Seoul 100-715, Korea; E-Mails: songwei@dongguk.edu (S.W.); khum@dongguk.edu (K.U.); 2 Division of Electronics and Electrical Engineering, Dongguk University-Seoul, 2 Pildong 3 Ga, Jung-gu, Seoul 100-715, Korea; E-Mail: cswon@dongguk.edu; 3 Agency for Defense Development, Bugyuseong daero 488 beon gi, Yoseong, Daejeon 305-152, Korea; E-Mail: sdsim@add.re.kr

**Keywords:** mobile robot, terrain reconstruction, multisensor integration, Gibbs-MRF, classification

## Abstract

Terrain classification allows a mobile robot to create an annotated map of its local environment from the three-dimensional (3D) and two-dimensional (2D) datasets collected by its array of sensors, including a GPS receiver, gyroscope, video camera, and range sensor. However, parts of objects that are outside the measurement range of the range sensor will not be detected. To overcome this problem, this paper describes an edge estimation method for complete scene recovery and complete terrain reconstruction. Here, the Gibbs-Markov random field is used to segment the ground from 2D videos and 3D point clouds. Further, a masking method is proposed to classify buildings and trees in a terrain mesh.

## 1. Introduction

Object segmentation and classification are widely researched topics in surveying, mapping, and autonomous navigation by mobile robots [1,2]. These techniques allow a robot to navigate through and interact with its environment by providing quickly accessible and accurate information regarding the surrounding terrain [3].

The multiple sensors mounted on such robots collect terrain information only in the form of three-dimensional (3D) point clouds and two-dimensional (2D) images [4]. Then object classification methods are applied to these datasets to classify salient features [5,6].

When mobile robots, especially ground-based autonomous robots, detect surrounding terrain information, some parts of objects are outside the measurement of range sensors. Therefore the classification will be incomplete and inaccurate. This incompleteness can be addressed with video cameras, which can provide terrain scenes with complete scenes in the far field. However, it is difficult to estimate objects' surfaces using only video cameras. Thus, datasets from a multiple sensors [7] must be integrated for a terrain classification system that allows accurate and reliable map annotation.

Here we propose a method of terrain classification, consisting of ground segmentation and building and tree classification, using complete scene recovery. We use 3D point clouds and 2D images for fast ground segmentation method using the Gibbs-Markov random field (MRF) method with a flood-fill algorithm. To recover complete scenes, we propose the Gibbs-MRF method that detects the boundary pixels between objects and background in order to recover the missing tops of objects.

Considering that trees have a porous surface and buildings have a uniform distribution, we classify buildings and trees based on the horizon spatial distribution using a masking method. Finally, the terrain classification results are used to create a 3D textured terrain mesh, which is compatible with global information database collection, semantic map generation, and augmented reality applications.

The present paper is organized as follows: in Section 2, we discuss related work on multisensor integration, interpolation, ground segmentation, and object classification in real-world applications. In Section 3, we describe our proposed framework for terrain reconstruction and object classification. In Section 4, we analyze the results of the proposed ground segmentation, height estimation, and object classification methods. In Section 5, we present our conclusions.

## 2. Related Work

Real-world reconstruction involves several sub-processes, including terrain mesh generation, interpolation, traversable region assessment, and object classification.

### 2.1. Multisensor Integration

To represent a robot's surrounding terrain in a virtual environment, it is necessary to reconstruct a terrain model using an integrated dataset obtained from multiple sensors [8–12]. Rovira-Más [13] proposed a density grid for 3D reconstruction from information obtained from stereo cameras, a localization sensor, and an inertial measurement unit. Sukumar [3] provided a convenient visualization method by integrating sensed datasets into a textured terrain mesh. However, it is difficult for these systems to process the large datasets obtained in outdoor environments and achieve on-line rendering.

Other researchers have enhanced the performance of terrain reconstruction to provide on-line photo-realistic visualization. Kelly [9] describes real-world representation methods using video-ranging modules. In the near field, 3D textured voxel grids are used to describe the surrounding terrain, whereas a billboard texture in front of the robot is used to show scenes in the far field. However, a range sensor cannot sense all terrain information, often leaving empty spaces in the terrain model in practice.

### 2.2. Interpolation in Empty Regions

Recovery of these “unsensed” regions plays a major role in obstacle avoidance. Some researchers apply interpolation algorithms to fill empty holes and smooth terrain [14–17]. For example, to estimate such unobserved data, Douillard [18] interpolates grids in empty regions in elevation maps in order to propagate label estimates. However, it is difficult to use these methods to recover missing information that is beyond the measurement range of the sensors.

Wellington [19] applies a hidden semi-Markov model to classify terrain vertical structure into ground, trees, and free space classes for each cell of a voxel-based terrain model. Then an MRF algorithm is used to estimate ground and tree height. However, this height estimation process simply averages across cells using neighbor data and cannot estimate actual height values.

In hardware design research, Früh [7] utilizes a vertical 2D laser scanner to measure large buildings and represent streetscapes in urban environments. When an object is located between the sensors and a building, some regions of the building cannot be sensed by the laser scanner as they are blocked by the object. These missing regions can be easily filled by planar or horizontal interpolation algorithm.

### 2.3. Traversable Region Segmentation

Ground segmentation is a widely studied topic necessary to determine the traversable regions in a terrain. Pandian [2] classifies terrain features into rocky, sandy, and smooth classes solely from 2D images. The segmented results take the form of a rectangular grid, instead of polygon shape. Therefore, this method lacks precision.

The MRF algorithm is effective in object segmentation from 2D images and 3D point clouds [20–26]. However, it is difficult to specify the probability density functions (PDF) in MRF. To solve this problem, the Hammersley-Clifford theorem proves an equivalence relationship between MRF and the Gibbs distribution [25]. However, computation of the Gibbs-MRF is too complicated for real-time ground segmentation.

### 2.4. Object Segmentation and Classification

Object segmentation is necessary to extract features, implement classification, and generate a semantic map. Weiss [27] utilizes a RANSAC algorithm to detect the ground and organize a point cloud into several clusters by segmenting plants and measuring plant positions. Segmented plants are estimated with high accuracy. However, this method can only be used for small plants, because it cannot be applied to objects outside the sensor's measurement range.

Golovinskiy [28] proposed a graph-based object segmentation method. The 3D points sensed by the range sensor are grouped into nodes of a graph using the k-nearest neighbor algorithm. The min-cut algorithm is then applied to segment the nodes into several objects. Lalonde [29] segments 3D points into scatter-ness, linear-ness, and surface-ness saliency features. In this method, an object model with a special saliency feature distribution is trained off-line by fitting a Gaussian mixture model (GMM) using the expectation-maximization (EM) algorithm. New data can be classified on-line into the model with a Bayesian classifier.

Huber [30] proposed a semantic representation method for building components. The floor and ceiling components are identified by finding the bottom-most and top-most local maxima in the height histogram. After low-density cells in the ground plane histogram are removed, the wall lines are detected using the Hough transform.

Nüchter [8] described a feature-based object detection method for 3D point cloud classification. First, the plans are extracted from the 3D point cloud using the RANSAC algorithm. Then, the wall, floor, ceiling, and other objects are labeled according to the defined scene interpretation. Finally, the objects are detected from a 2D image taken from the 3D rendering result.

In this paper, we discuss a multisensor integration method. For ground segmentation, we use the Gibbs-MRF and a flood-fill algorithm. Further, in contrast to interpolation methods, we propose a height estimation algorithm to recover unsensed regions, especially for objects at a height and outside the sensor's range of measurement.

## 3. Terrain Reconstruction and Object Classification

**Figure 1.**
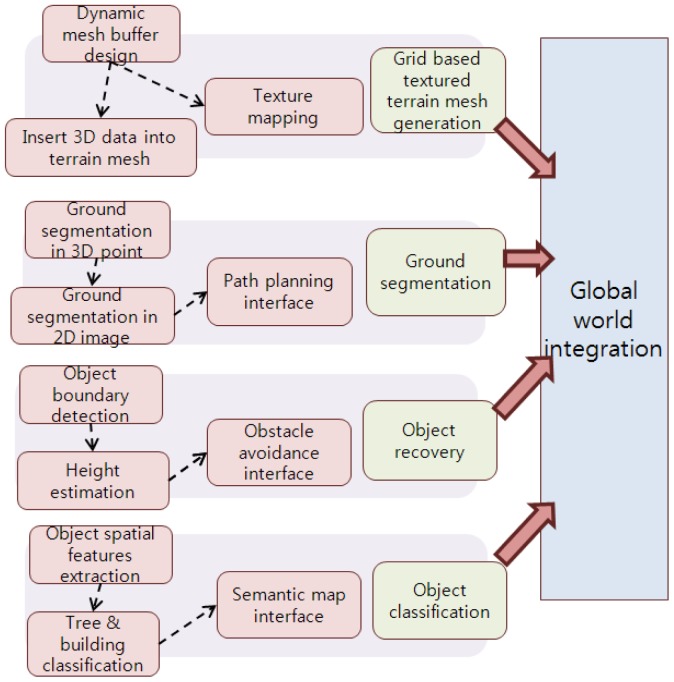
Framework for outdoor terrain reconstruction and object classification.

We describe a framework for outdoor terrain reconstruction and object classification, as shown in Figure 1. The integrated sensors provide a dataset of 2D images, 3D point clouds, and mobile robot navigation information. We integrate these dataset into a grid-based textured terrain mesh. Then, we describe a ground segmentation method that identifies the features such as the ground, obstacles, and the background.

As mentioned, for objects partially beyond the range of detection of the range sensor, we propose a height estimation method to recover the complete scene in the terrain mesh. Finally, we classify the objects into buildings and trees on the basis of their spatial distribution.

### 3.1. Grid-Based Textured Terrain Mesh

**Figure 2.**
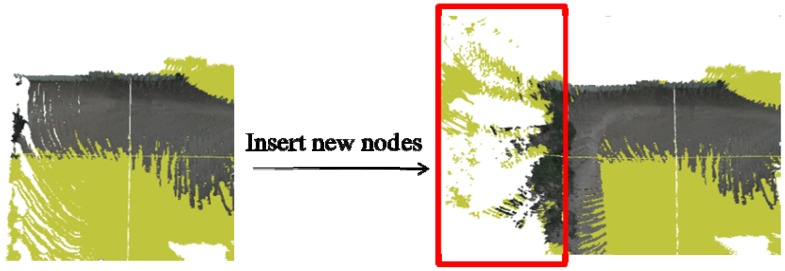
Grid-based ground modeling.

We integrate the sensed dataset into a grid-based textured terrain mesh. First, we project the 3D points onto the 2D image in front of the robot and get a coordinate in 2D image, named UV vector, for each 3D point. Then, we transform the local 3D points into global coordinates, and register them on the terrain mesh. The terrain mesh is generated using several grids, each with 151 × 151 textured vertices. In this application, the cell size is 0.125 × 0.125 m^2^. The height value of each cell is updated with the registered 3D points. If a new 3D point is to be inserted into the reconstructed terrain mesh but is outside the existing grids, we create a new grid to register this point, as shown in Figure 2.

After registration of all points, a ground segmentation algorithm is implemented to segment ground data and non-ground data in the 2D image. Then, a height estimation method is used to recover the missing regions outside the sensor's measurement range. Finally, the tree and building objects are classified using a classification operation. In our implementation, the user controls a virtual camera to study the reconstructed terrain from different viewpoints. A virtual robot model is loaded on the terrain to show the robot's navigation information in the real world.

### 3.2. Ground Segmentation Method

We classify each pixel in the 2D image into ground and non-ground classes on the basis of the probability of it being in that configuration, which depends on its connected neighbors. Therefore, we can apply the MRF for ground segmentation. However, it is difficult to determine the probability because it must be computed from local and neighbor observations. According to the Hammersley-Clifford theorem, we can solve this problem using the Gibbs-MRF model.

Given observation *d* and configuration *f*, we find the best possible configuration f* for site *s* using the following optimum solution:

(1) $$f^{*}(s)=\arg\underset{f}{\max}p(X_{s}=f|X_{t}=d,\forall t\neq s)$$

The probability of a site's configuration is calculated using the Gibbs distribution [22]:

(2) $$p(f)=Z^{-1}e^{-\frac{1}{T}U(f)}$$

(3) $$U(f)=\sum_{c\in C}V_{c}(f)$$

(4) $$Z=\sum_{f}e^{-\frac{1}{T}U(f)}$$

We define a clique as a neighboring set, and a clique set *C* as a collection of single-site and pair-site cliques. A potential function *V_c_*(*f*) is defined to evaluate the effect of neighbor sites in clique *c*.

According to the Bayes' rule, the solution of Equation (1) is as follows:

(5) $$f^{*}=\arg\underset{f}{\max}p(f|d)=\arg\underset{f}{\max}p(d|f)p(f)=\arg\underset{f}{\min}U(f|d)=\arg\underset{f}{\min}\{U(d|f)+U(f)\}$$

The energy function of *U*(*d*|*f*) + *U*(*f*) is defined to evaluate the effect of the neighbor sites in single-site and pair-site potential cliques, as follows:

(6) $$U(d|f)+U(f)=\sum_{s\in C_{1}}V_{1}(f_{s})+\sum_{\{s,s'\}\in C_{2}}V_{2}(f_{s},f_{s'})+\sum_{s\in C_{1}}V_{1}(d_{s}|f_{s})+\sum_{\{s,s'\}\in C_{2}}V_{2}(d_{s},d_{s'}|f_{s},f_{s'})$$

The evaluations of the clique potential functions *V*_1_(*f_s_*) and *V*_1_(*d_s_*|*f_s_*) depend on the local configuration and observations of clique *C*_1_. The clique potential functions *V*_2_(*f_s_*, *f_s'_*) and *V*_2_(*d_s_*, *d_s'_*|*f_s_*, *f_s'_*) are evaluations of the pair-site consistency of clique *C*_2_.

**Figure 3.**
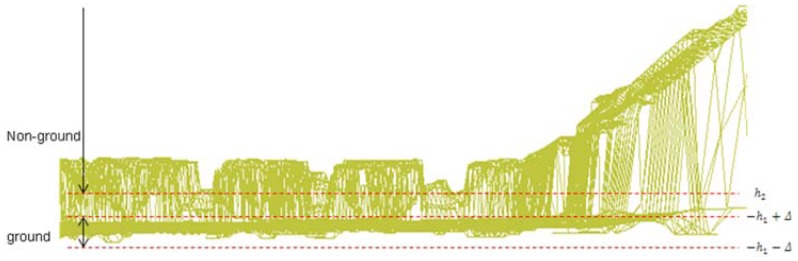
Rough segmentation of 3D ground data.

When we apply the Gibbs-MRF to ground segmentation in a 2D image, we first determine a set of pixels whose configurations are in the ground class with high confidence. We initially segment the 3D points as ground data using the robot vehicle's height *h*_1_ as the standard. We assume that if the y coordinate of a 3D point is ranging from −*h*_1_ − Δ to −*h*_1_ + Δ, then this point is ground data, as shown in Figure 3. This step is a rough ground segmentation process, which produces a dataset *G*_1_.

**Figure 4.**
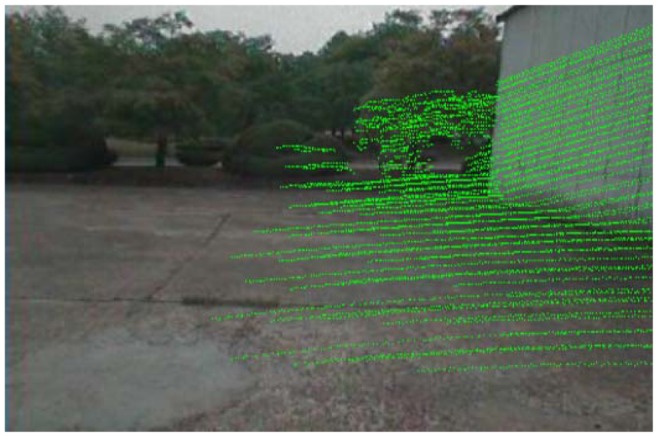
Projection results as green pixels in an image.

Then we find the projected pixels in the 2D image from the points in *G*_1_, using the projection matrix as follows:

(7) t=KR[I|−Cam]T

where the homogeneous coordinates of image pixel *t* are projected from the homogeneous coordinates of the 3D point *T*. Cam is defined as the vector of the camera's position, the matrix *R* is defined as the mobile rotation matrix, and *I* is an identity matrix. The camera calibration matrix *K* is defined as follows:

(8) $$K=\left[\begin{array}{ccc} l & 0 & p_{x}\\ 0 & l & p_{y}\\ 0 & 0 & 1 \end{array}\right]$$

where *l* is the focal length of the camera, and the 2D coordinate (*p_x_*, *p_y_*) is the center position of the captured image. As shown in Figure 4, the 2D pixel dataset $G_{1}^{'}$ is mapped from the dataset *G*_1_. We determine the configuration of site $s\in G_{1}^{'}$ as ground.

We apply the Gibbs-MRF algorithm to classify the configurations of other pixels into the ground or non-ground classes. We consider that:

- If the configuration of site *s* is same as its observation, the probability of this configuration is high.
- If the configuration of site *s* is same as the configuration of its neighboring site *s'*, the probability of this configuration is high.
- If the configuration of site *s* is same as the configuration of its neighboring site *s'*, and the difference between these observations *d_s_* and *d_s_*_′_ is low, the probability of this configuration is high.

The clique potential functions are formulated as follows:

(9) $$V_{1}(f_{s})=\{\begin{array}{cccc} -\alpha & if(s\in G_{1} & \text{and} & f_{s}=\text{ground})\\ & or(s\notin G_{1} & \text{and} & f_{s}=\text{nonground})\\ +\alpha & if(s\notin G_{1} & \text{and} & f_{s}=\text{ground})\\ & or(s\in G_{1} & \text{and} & f_{s}=\text{nonground}) \end{array}$$

(10) $$V_{1}(d_{s}|f_{s})=\{\begin{array}{cc} -\alpha & if(d_{s}=f_{s})\\ +\alpha & if(d_{s}\neq f_{s}) \end{array}$$

(11) $$V_{1}(f_{s},f_{s'})=\{\begin{array}{cc} -\beta & if(f_{s}=f_{s})\\ +\beta & if(f_{s}\neq f_{s}) \end{array}$$

(12) $$V_{1}(d_{s},d_{s'}|f_{s},f_{s'})=\{\begin{array}{cc} -\gamma e^{-\|d_{s}-d_{s'}\|} & if(f_{s}=f_{s'})\\ +\gamma e^{-\|d_{s}-d_{s'}\|} & if(f_{s}\neq f_{s'}) \end{array}$$

Here, the constants *α*, *β*, and *γ* are positive numerical values. The configuration *f_s_* depends on whether the pixel *s* belongs to the ground dataset $G_{1}^{'}$. The formula ‖*d_s_* – *d_s'_*‖ is defined as the color difference between observations *d_s_* and *d_s'_*.

We derive Equation (5) using the potential functions defined in Equations (9–12), and label the configuration of each pixel.

To reduce the computation load of Gibbs-MRF, we apply a flood-fill algorithm to compute the configurations of pixels inside the boundary between ground and non-ground. The pseudocode for ground segmentation using the flood-fill algorithm is as follows:
for each site *s* in 
$G_{1}^{'}$ configuration *f*(*s*) = ground; enqueue neighbour sites of *s* into a queue Q; while (Q is not empty)  dequeue a site *s′* from the Q;  if (f* (*s*′) =ground)   enqueue neighbor sites of *s′* into Q;  endif; endwhile;endfor;

Starting with the pixel set $G_{1}^{'}$, we estimate the configurations of the neighboring pixels. We apply the Gibbs-MRF algorithm to classify the configurations of other pixels into the ground or non-ground classes.

The pixels with a ground configuration are grouped into dataset $G_{2}^{'}$, which is shown as the blue region. The other regions contain objects and background textures. We classify the ground vertices in the 3D terrain mesh, which are mapped to the pixels in the dataset $G_{2}^{'}$, as shown in Figure 11(b).

### 3.3. Complete Scene Recovery

**Figure 5.**
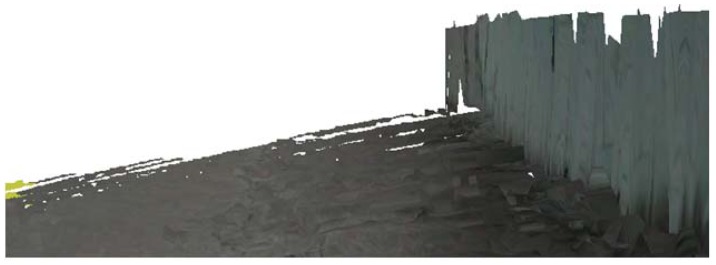
Terrain reconstruction results.

When mobile robots detect surrounding terrain information, some parts of objects are outside the measurement of range sensors. We see that the top of the building is missing in the terrain reconstruction result, shown as Figure 5.

We propose a height estimation method to solve the problem of missing regions by estimating the y coordinate of an object's top boundary.

**Figure 6.**
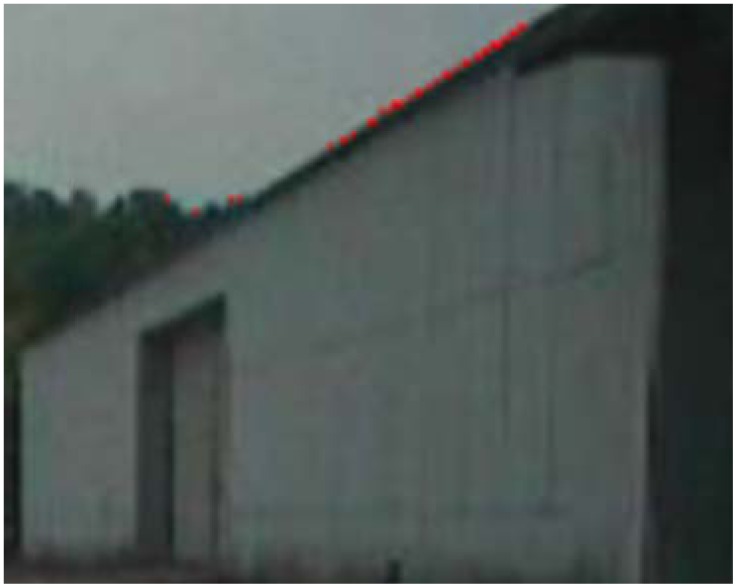
Boundary searching in 2D image.

Using the ground data segmentation result, we assume that the non-ground vertices in the terrain mesh belong to objects, because background data, such as the sky, cannot be sensed by the range sensor. Next, we project these vertices onto pixels in a 2D image, whose configuration is determined as being part of an object. We apply the Gibbs-MRF method to classify the non-ground pixels into objects and background classes, in order to detect the boundary pixels between objects and background. The boundary detection results are shown as red pixels in Figure 6.

**Figure 7.**
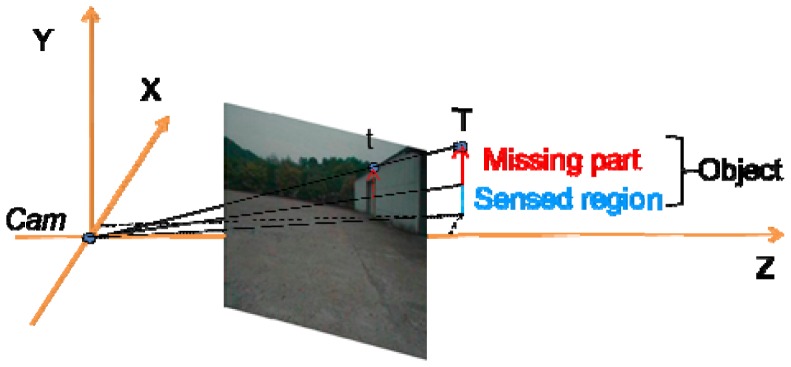
Height estimation process.

**Figure 8.**
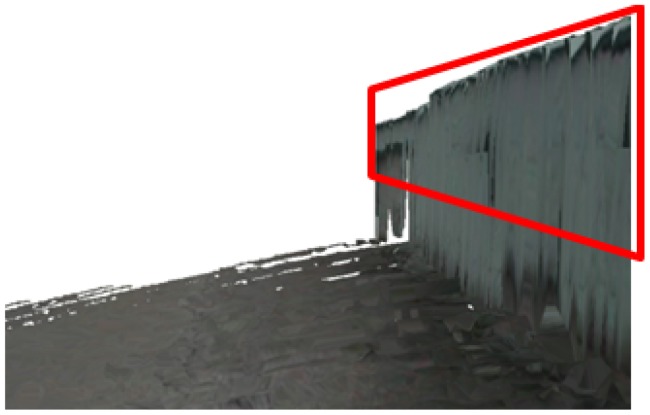
Reconstruction result after complete scene recovery. The recovered parts are indicated by the red rectangle.

We find the boundary's y coordinates using an inverse process of projection from 2D pixels to 3D points. We place the camera centre at the origin. The projection ray from the origin to the object vertex gives an estimate of the height of that object vertex, as shown in Figure 7. Because the horizon coordinates of the 3D object vertex in the terrain mesh are fixed, we update the elevation value of each object vertex in the terrain mesh to obtain the results shown in Figure 8.

### 3.4. Building and Tree Classification

**Figure 9.**
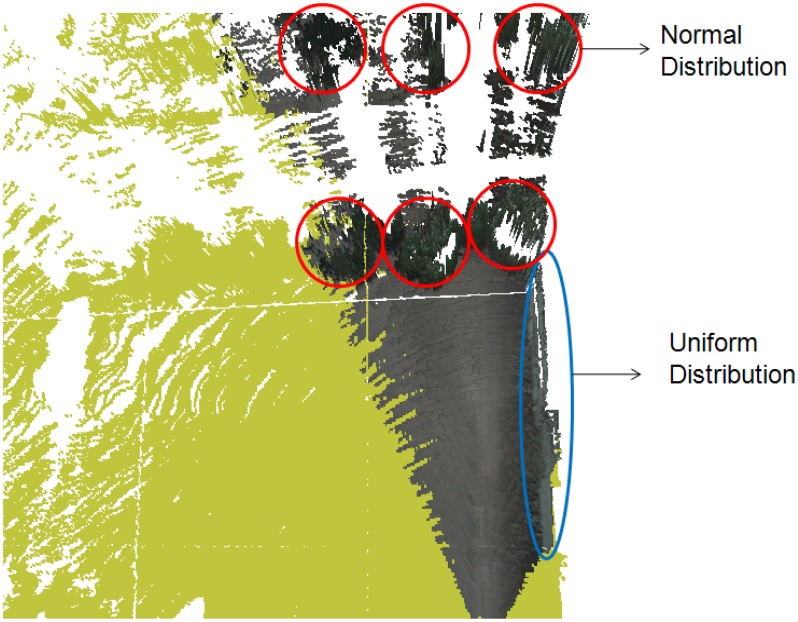
Spatial distributions of buildings and trees.

**Figure 10.**
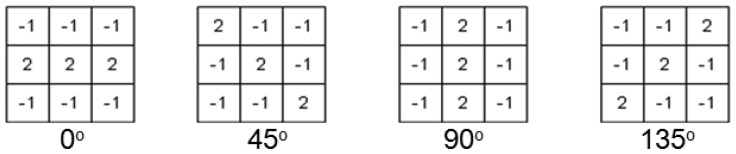
Line detector masks.

We consider tree objects, including both grass and trees, to have a porous surface that allows rays from the range finder to pierce through to the inside. This is in contrast to buildings, for which the 3D range finder only detects points on the outer surface. Therefore, the horizon shape of a building has a uniform distribution, whereas that for a tree has a normal distribution. As shown in Figure 9, we can see that the horizon structure of the buildings consists of the line-like components. We classify buildings by detecting these lines using the masks described in Figure 10.

The convolution function for the masking method is:

(13) $$U(i,j)=\sum_{n=-s}^{s}\sum_{m=-s}^{s}h(i-m,j-n)f(m,n)$$

where *h*(*i*, *j*) is the elevation value of a vertex in the terrain mesh, *f*(*m*, *n*) is the value in a mask cell, and s is the size of the mask. If *U*(*i*, *j*) is larger than a threshold, we determine the vertex (*i*, *j*) belongs to a building. If not, we determine the vertex belongs to a tree. After classifying buildings in the terrain mesh, we map the building vertices onto the 2D images in order to identify the sensed buildings in the 2D images.

## 4. Experiments

Experiments were carried out using a mobile robot with integrated sensors, including a GPS receiver, gyroscope, video camera, and range sensor. We utilized HDL-32E Velodyne sensor to scan 3D points in an unknown environment. It provides approximately 694,292 laser shots per second. The Valid Data Range is approximately 70 m. The proposed algorithms were implemented by the laptop with an 2.82 GHz Intel(R) Core(TM)2 Quad CPU, a GeForce GTX 275 graphics card and 4 GB RAM. We drove the robot around an outdoor area of 104 square meters, including buildings and trees. The upper parts of these objects were outside the range of sensor, but were captured in the 2D images.

**Figure 11.**
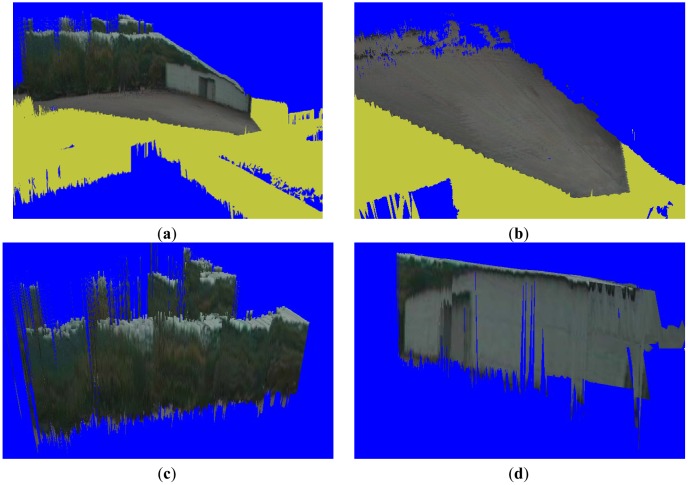
Segmentation and classification results. (**a**) Complete scene recovery. (**b**) Ground segmentation in the terrain mesh. (**c**) Tree classification in the terrain mesh. (**d**) Building classification in the terrain mesh.

The final terrain classification result, as shown in Figure 11, is obtained in five steps: first, we reconstruct a textured terrain mesh in a virtual environment by integrating the packages. Then, we segment the ground vertices in the terrain mesh and map them onto 2D pixels. Next, we segment all the ground pixels using the Gibbs-MRF model with the flood-fill algorithm. Then, we estimate object boundaries in the 2D images using the object vertices in the terrain mesh and evaluate the height of each object cell in the terrain mesh. Finally, we classify buildings and trees in the terrain mesh based on the proposed masking method. Because we classify building objects in x-z plane, some pixels of trees exist above the buildings in Figure 11(d).

We discuss the ground segmentation results by using a confusion matrix, which is shown in Table 1. The *ground* and *non* – *ground* rows represent the actual ground and non-ground classes respectively. The $\bar{\text{ground}}$ and $\bar{\text{non}-\text{ground}}$ columns represent the inferred ground and non-ground classes respectively.

We segment the ground data in a 2D image with 512 × 256 pixels. The confusion matrix is computed by a supervised method. We group the pixels into ground and non-ground classes manually. If an actual ground pixel is grouped under the ground class, the inferred class $\bar{\text{ground}}$ increases by one. If not, $\bar{\text{non}-\text{ground}}$ increases by one.

Table 1 indicates that 97.68% of the ground region has been segmented on average. The ratio of the inferred errors to the actual classes, including ground and non-ground, is 3.19% on average.

**Figure 12.**
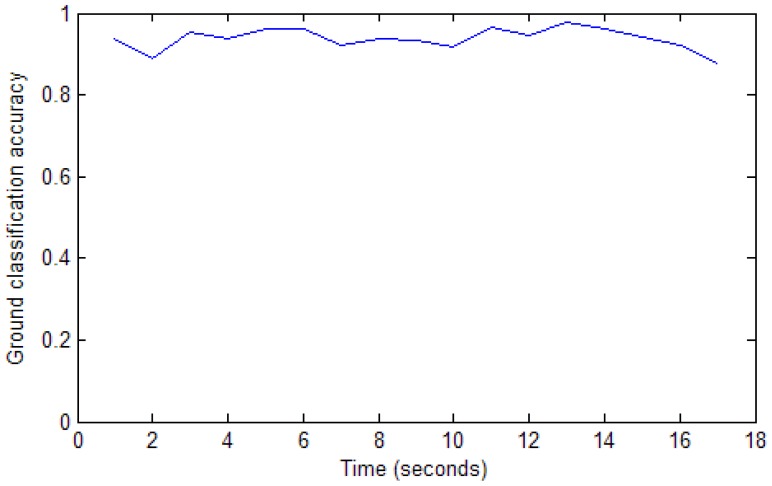
Ground classification accuracy.

We implement the ground segmentation in 2D image every second. Figure 12 shows the ground classification accuracy samples during the robot navigated in an unknown environment. The accuracy value is calculated as the ratio between the inferred ground pixels and the actual ground pixels.

**Figure 13.**
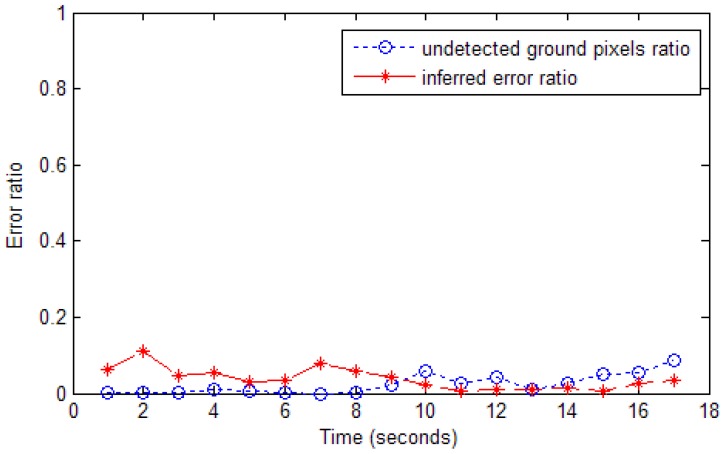
Errors in ground classification.

We define two types of classification errors in this project. One of them results from undetected ground pixels. If ground pixels are inferred as non-ground pixels, we define them as inferred errors. Figure 13 shows samples of undetected ground pixel ratio and inferred error ratio.

**Figure 14.**
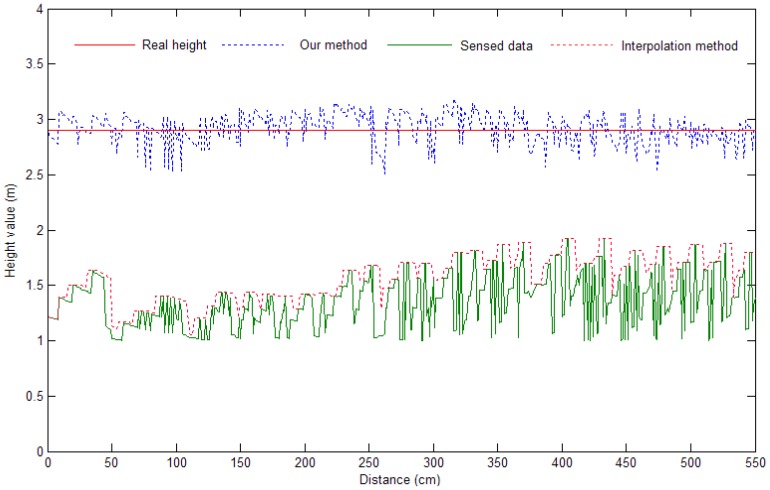
Height estimation result.

We then detect the edge of objects by using the non-ground classification result. We investigate the performance of the proposed height estimation method by comparing the obtained values with the actual heights (2.90 m on average). Since the range sensor scans objects only up to a height of 1.8 m, the upper parts of buildings cannot be sensed. However, as shown in Figure 14, we recover the missing parts from the incomplete terrain mesh, and the average estimated height value is 2.92 ± 0.11 m. In Figure 14, the *x*-axis represents the distance between the estimated vertices with the first estimated vertex.

The previously proposed interpolation algorithms average the empty region using the surrounding 3D points. These methods do not recover the actual shape of the unsensed region. However, using our proposed height estimation method, we successfully recover the actual shape of the missing parts.

**Figure 15.**
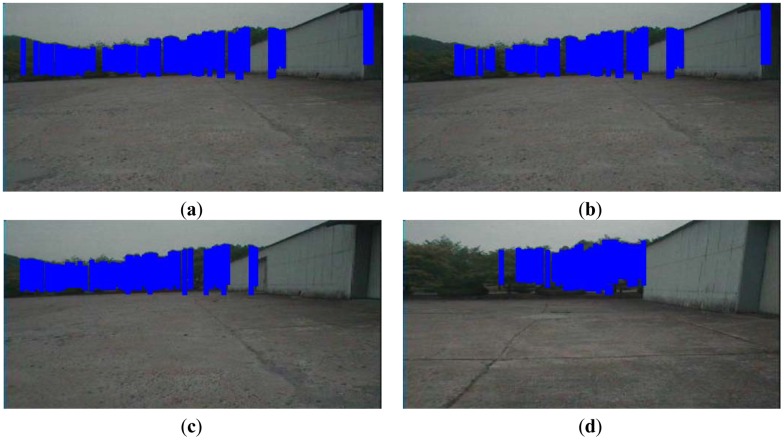
Tree classification results: (**a**) frame 50; (**b**) frame 100; (**c**) frame 200; and (**d**) frame 800.

We render the textured terrain mesh and represent the texture of the ground, trees, and buildings at an average of 11.43 frames per second (FPS) using the Gibbs-MRF model along with the flood-fill algorithm. This is faster than the case where only the Gibbs-MRF model is used (8.37 FPS). After recovering complete scenes in the terrain mesh, we classify objects into tree and building classes. The tree classification results are indicated in blue color in the 2D images in Figure 15. In the 50th and 100th frames, the objects are located far from the robot, so that noise exists in the sensed objects, especially at the corners. When the robot moves closer to the building in the 200th frame, the corner shape is detected accurately. The corner pixels are grouped in the building class. When the robot is located near the trees in the 800th frame, the accuracy of the range sensor is higher than that when the robot is far from the trees. Finally, the noise in the spaces between the trees is removed in the reconstructed terrain mesh.

We use a manual supervised method to classify the pixels in the 2D images of Figure 15(a–d) into tree and building objects. By using the inferred results and through manual classification, the confusion matrices in Tables 2–5 are obtained. When the robot moves closer to the objects, the spatial distribution of the objects is detected with low noise; in this case, object classification is performed correctly and the error ratio is low.

## 5. Conclusions

This paper described a method of effective segmentation of ground, buildings, and trees for automated surveying and mapping by mobile robots. The method was found effective in an outdoor environment for a mobile robot with a range sensor, video camera, GPS receiver, and a gyroscope.

The complete shape of objects that are partly outside sensors' range of measurement is accurately recovered. The accurate height estimation allowed successful classification of buildings and trees on the basis of their spatial distribution. However, the height estimation algorithm does not work well for recovering the buildings which are not uniform in color or have overhanging roofs. In future, we will improve the system to deal with these problems.

## Figures and Tables

**Table 1.** Ground segmentation performance.

**Truth/Inferred**	$\bar{\text{ground}}$	$\bar{\text{non}-\text{ground}}$
*ground*	67977	1611
*non* – *ground*	2580	58904

**Table 2.** Tree classification result for Figure 15(a).

**Truth/Inferred**	$\bar{\text{tree}}$	$\bar{\text{building}}$
*tree*	9918	3026
*building*	2828	10235

**Table 3.** Tree classification result for Figure 15(b).

**Truth/Inferred**	$\bar{\text{tree}}$	$\bar{\text{building}}$
*tree*	11983	3106
*building*	1749	9847

**Table 4.** Tree classification result for Figure 15(c).

**Truth/Inferred**	$\bar{\text{tree}}$	$\bar{\text{building}}$
*tree*	11536	905
*building*	1238	14652

**Table 5.** Tree classification result for Figure 15(d).

**Truth/Inferred**	$\bar{\text{tree}}$	$\bar{\text{building}}$
*tree*	17196	33
*building*	340	17771
